# A new standard nomenclature for proteins related to Apx and Shroom

**DOI:** 10.1186/1471-2121-7-18

**Published:** 2006-04-14

**Authors:** Olivier Hagens, Andrea Ballabio, Vera Kalscheuer, Jean-Pierre Kraehenbuhl, M Vittoria Schiaffino, Peter Smith, Olivier Staub, Jeff Hildebrand, John B Wallingford

**Affiliations:** 1 Dept. of Human Molecular Genetics, Max Planck Institute for Molecular Genetics, Berlin, Germany; 2 Telethon Institute of Genetics and Medicine, Naples, Italy; 3 Swiss Institute for Experimental Cancer Research and the Institute of Biochemistry, University of Lausanne, Lausanne, Switzerland; 4 Dept. of Biotechnology, San Raffaele Scientific Institute, Milan, Italy; 5 Dept. of Physiology and Biophysics, University of Alabama at Birmingham, Birmingham, AL, USA; 6 Dept. of Pharmacology & Toxicology, University of Lausanne, Lausanne, Switzerland; 7 Dept. of Biological Sciences, University of Pittsburgh, Pittsburgh, PA, USA; 8 Dept. of Molecular Cell and Developmental Biology & Institute for Cellular and Molecular Biology, University of Texas, Austin, TX, USA

## Abstract

Shroom is a recently-described regulator of cell shape changes in the developing nervous system. This protein is a member of a small family of related proteins that are defined by sequence similarity and in most cases by some link to the actin cytoskeleton. At present these proteins are named Shroom, APX, APXL, and KIAA1202. In light of the growing interest in this family of proteins, we propose here a new standard nomenclature.

In 1992, the primary structure of an apical protein in *Xenopus *(Apx) was described [1]. Since then, three related proteins have been characterized, namely the human proteins APXL (apical protein *Xenopus*-like) [2] and KIAA1202 [3] and mouse Shroom [4], named after the mouse mutant phenotype. We now know that the Apx protein of *Xenopus *is not in fact the orthologue of human APXL. Instead, the protein previously called human APXL2 is the likely homologue of frog Apx, while human APXL is the likely homologue of a *Xenopus *APXL. In this letter, we report a new standardized nomenclature to eliminate the confusing present naming situation for these proteins (Table 1).

**Table 1:** New nomenclature for Shroom-related proteins

**GenBank Accession Number**	**Previous name**	**New name**
CAA78718	*X. laevis *Apx	xShroom1
NP_597713	*H. sapiens *APXL2	hShroom1
CAA58534	*H. sapiens *APXL	hShroom2
ABD19518	*M. musculus *Apxl	mShroom2
AAF13269	*M. musculus *ShroomL	mShroom3a
AAF13270	*M. musculus *ShroomS	mShroom3b
NP_065910	*H. sapiens *Shroom	hShroom3
ABD59319	*X. laevis *Shroom-like	xShroom3
NP_065768	*H. sapiens *KIAA1202	hShroom4a
AAK95579	*H. sapiens *SHAP-A	hShroom4b
DQ435686	*M. musculus *KIAA1202	mShroom4
ABA81834	*D. melanogaster *Shroom	dmShroom
EAA12598	*A. gambiae *Shroom	agShroom
XP_392427	*A. mellifera *Shroom	amShroom
XP_783573	*S. purpuratus *Shroom	spShroom

From global multiple alignments of genomic sequences, it is clear that these proteins are not simply encoded by homologous genes. There are in fact four different proteins in this family, showing similarity in their domains (Table 2), which include a PDZ and two Apx/Shrm domains (ASD1 and ASD2) and putative EVH1 and PDZ binding sites [4]. It should be noted however that Apx lacks the PDZ domain and the EVH1 binding site, APXL lacks a PDZ binding site and KIAA1202 does not contain an obvious ASD1 domain. Therefore, the ASD2 domain seems to be the common denominator among family members.

**Table 2:** Sequence identity matrix for the four different Shroom proteins which have been characterised experimentally.

Shroom^a^	1	2	3	4a
1	100/NA/100/100^b^	11.7/NA/32.9/37.7	10.9/NA/29.8/32.9	9.5/NA/NA/35.3
2		100/100/100/100	25.4/67.5/44.6/68.2	20.1/61.0/NA/65.8
3			100/100/100/100	15.9/63.6/NA/61.6
4a				100/100/NA/100

^a ^This table makes use of the new nomenclature presented in Table 1. To avoid evolution-based dissimilarity, the human homologues have been used in the analysis. ^b ^Percent sequence identity is given in the format global/PDZ/ASD1/ASD2; NA, not applicable. Global sequence identity is based on those residues aligning to hShroom1 residues 1 – 826. The alignments on which this matrix is based were created using ClustalW. They are available upon request.

Bioinformatics-based searches identified Shroom-related proteins in all chordates examined. In addition, insect genomes, including *Drosophila melanogaster*, *Anopheles gambiae *and *Apis mellifera*, encode a partially related protein containing an ASD2 domain (Table 1). Finally, BLAST searches of the deposited sequences from invertebrate genome projects identify what may be considered Shroom orthologues in both *Ciona intestinalis *(data not shown) and *Strongylocentrotus purpuratus *(Table 1). Based on the putative open reading frames and genomic organization, these predicted proteins contain, at least, the N-terminal PDZ domain and the C-terminally positioned ASD2 motif.

To clarify future studies, we propose a unifying nomenclature, emphasizing the relatedness of those proteins (Table 1). We feel that while the founding member is Apx, this name is undesirable as a root for naming this family because it requires that '*Xenopus*' would appear in protein names from all species. Instead, we propose that the new nomenclature be based upon the name 'Shroom' as this is now the most thoroughly studied member of the family [4-6]. An Arabic number following 'Shroom' would distinguish between the different proteins. A lower-case letter would distinguish between different protein products encoded by the same locus generated by alternative mRNA processing. According to these rules, we suggest the re-naming presented in Table 1.

Several papers suggest that these related proteins play diverse and important roles in the development of the nervous system and other tissues [2-8]. Future studies will be required to show if sequence similarity among Shroom protein family members is mirrored by conservation of their cellular and molecular function.
